# Supplementary material for: Exploring the multiple policy objectives for primary care networks: a qualitative interview study with national policy stakeholders
Source: BMJ Open. 2020 Jul 5;10(7):e038398. doi: 10.1136/bmjopen-2020-038398 (PMC7337885; doi:10.1136/bmjopen-2020-038398)
Supplement: Author's manuscript [file bmjopen-2020-038398.draft_revisions.pdf]

BMJ Open

BMJ Open is committed to open peer review. As part of this commitment we make the peer review history of every article we publish publicly available.

When an article is published we post the peer reviewers' comments and the authors' responses online. We also post the versions of the paper that were used during peer review. These are the versions that the peer review comments apply to.

The versions of the paper that follow are the versions that were submitted during the peer review process. They are not the versions of record or the final published versions. They should not be cited or distributed as the published version of this manuscript.

BMJ Open is an open access journal and the full, final, typeset and author-corrected version of record of the manuscript is available on our site with no access controls, subscription charges or pay-per-view fees (http://bmjopen.bmj.com).

If you have any questions on BMJ Open's open peer review process please email info.bmjopen@bmj.com

BMJ Open

Exploring the multiple policy objectives for Primary Care Networks: a qualitative interview study with national policy stakeholders

Journal:	*BMJ Open*
Manuscript ID	bmjopen-2020-038398
Article Type:	Original research
Date Submitted by the Author:	09-Mar-2020

Complete List of Authors:	Checkland, Kath; The University of Manchester, Division of Population Health, Health Services Research, and Primary Care; School of Health Sciences; Faculty of Biology, Medicine and Health<br>Hammond, Jonathan; University of Manchester, Division of Population Health, Health Services Research, and Primary Care; School of Health Sciences; Faculty of Biology, Medicine and Health<br>Warwick-Giles, Lynsey; The University of Manchester, Division of Population Health, Health Services Research, and Primary Care; School of Health Sciences; Faculty of Biology, Medicine and Health<br>Bailey, Simon; University of Kent, Centre for Health Services Studies

Keywords:	Health policy < HEALTH SERVICES ADMINISTRATION & MANAGEMENT, QUALITATIVE RESEARCH, Organisation of health services < HEALTH SERVICES ADMINISTRATION & MANAGEMENT, PRIMARY CARE

SCHOLARONE™
Manuscripts

I, the Submitting Author has the right to grant and does grant on behalf of all authors of the Work (as defined in the below author licence), an exclusive licence and/or a non-exclusive licence for contributions from authors who are: i) UK Crown employees; ii) where BMJ has agreed a CC-BY licence shall apply, and/or iii) in accordance with the terms applicable for US Federal Government officers or employees acting as part of their official duties; on a worldwide, perpetual, irrevocable, royalty-free basis to BMJ Publishing Group Ltd ("BMJ") its licensees and where the relevant Journal is co-owned by BMJ to the co-owners of the Journal, to publish the Work in this journal and any other BMJ products and to exploit all rights, as set out in our licence.

The Submitting Author accepts and understands that any supply made under these terms is made by BMJ to the Submitting Author unless you are acting as an employee on behalf of your employer or a postgraduate student of an affiliated institution which is paying any applicable article publishing charge ("APC") for Open Access articles. Where the Submitting Author wishes to make the Work available on an Open Access basis (and intends to pay the relevant APC), the terms of reuse of such Open Access shall be governed by a Creative Commons licence – details of these licences and which Creative Commons licence will apply to this Work are set out in our licence referred to above.

Other than as permitted in any relevant BMJ Author's Self Archiving Policies, I confirm this Work has not been accepted for publication elsewhere, is not being considered for publication elsewhere and does not duplicate material already published. I confirm all authors consent to publication of this Work and authorise the granting of this licence.

For peer review only

Exploring the multiple policy objectives for Primary Care Networks: a qualitative interview study with national policy stakeholders

Authors
Kath Checkland, PhD, Professor of Health Policy and Primary Care, GP
Centre for Primary Care and Health Services Research
Division of Population Health, Health Service Research & Primary Care
School of Health Sciences
Faculty of Biology, Medicine and Health
The University of Manchester
Williamson Building, Oxford Road, Manchester, M13 9PL
Email: katherine.h.checkland@manchester.ac.uk
Twitter: @khcheck

Jonathan Hammond, PhD
Centre for Primary Care and Health Services Research
Division of Population Health, Health Service Research & Primary Care
School of Health Sciences
Faculty of Biology, Medicine and Health
The University of Manchester
Williamson Building, Oxford Road, Manchester, M13 9PL

Lynsey Warwick-Giles, PhD
Centre for Primary Care and Health Services Research
Division of Population Health, Health Service Research & Primary Care
School of Health Sciences
Faculty of Biology, Medicine and Health
The University of Manchester
Williamson Building, Oxford Road, Manchester, M13 9PL

Simon Bailey, PhD
Centre for Health Services Studies
University of Kent
Canterbury, Kent, CT2 7NF

For peer review only

Abstract

Objectives
Primary Care Networks (PCNs) are voluntary incentivised collaborations between GP practices in the English NHS. They are key to delivering NHS Long Term Plan aspirations, and most GP practices have joined one. The study's aim was to explore national policy objectives underpinning PCNs, and mechanisms expected to help achieve these, from the perspective of those driving the policy.

Design
Qualitative semi-structured interviews and policy document analysis.

Setting and participants
National level policy maker and stakeholder interviewees (n=16). Policy document analysis of Network Contract Direct Enhanced Service draft service specifications.

Analysis
Interviews were transcribed, coded, and organised thematically according to policy objectives and mechanisms. Thematic data was organised into a matrix to allow prominent elements to be identified and emphasised accordingly. Themes were considered alongside objectives embedded in PCN draft service delivery requirements.

Results
Three themes of policy objectives and associated mechanisms were identified: (1)Supporting general practice; (2)Place-based inter-organisational collaboration; (3)Primary care 'voice'. Interviewees emphasised and sequenced themes differently suggesting meeting the objectives for one was necessary to realise another. Interviewees most closely linked to primary care emphasised the importance of (1). The objectives embedded in draft service delivery requirements primarily emphasised (2).

Conclusions
These policy objectives are not mutually exclusive but may imply different approaches to prioritising investment or necessitate more explicit temporal sequencing, with the stabilisation of a struggling primary care sector probably needing to occur before meaningful engagement with other community service providers can be achieved or a 'collective voice' is agreed. Multiple objectives create space for stakeholders to feel dissatisfied when implementation details do not match expectations, as the negative reaction to draft service delivery requirements illustrates. Our study offers policy makers suggestions about how confidence in the policy might be restored by crafting delivery requirements so all groups see opportunities to meet favoured objectives.

Keywords
health policy; qualitative research; organisation of health services; primary care

For peer review only

Strengths and limitations of this study

- Primary Care Networks represent a significant policy development in England, and we offer the first systematic analysis of national policy objectives as articulated by a range of different stakeholders.
- We interviewed 16 national level policy makers or stakeholders working in a range of organisations, including NHS England and NHS Improvement, and in government in the Department of Health and Social Care.
- National health care policy objectives are rarely subject to critical academic attention in the early stages of policy implementation, but doing so provides scope for better understanding challenges a policy may face and developing strategies to address these.
- This is a fast moving policy area and our results inevitably reflect a particular snapshot of time.

Main text: 4218 words

For peer review only

Introduction

Primary care in the UK is in crisis. Young doctors are not entering the speciality in sufficient numbers to cope with demand, older GPs are leaving in response to that rising demand(1, 2). The problem is complex, arising from the intersection between demographic change, rising population expectations of what services should deliver, and funding growth which has not matched the rise in costs(3, 4). In keeping with this complexity, potential solutions are multiple, with, for example, the GP Forward View(5) offering a number of potential policy fixes. Most recently, consensus has gathered around the idea that GP practices – traditionally independent contractors to the NHS – should work together more effectively to support one another and provide a broader range of services(6). To enable this, a centrepiece of the new NHS Long Term Plan(7) is the creation and funding of Primary Care Networks (PCNs). The underlying idea is relatively simple: incentivise practices to combine together into groups, so they can find economies of scale, employ a wider range of staff, link more effectively with community-based providers and improve services to patients.

Collaborations between GP practices are not new, in the UK or elsewhere. Internationally a ‘polyclinic’ model is said to offer the advantages of person-centred primary care alongside an extended range of services from a multidisciplinary team(9-11). In the UK, practices have voluntarily formed themselves into collaborative groups to undertake collective audits(12), deliver out-of-hours services(13) and support commissioning(14). Outcomes from these previous collaborations have been mixed. The ‘polyclinic’ model struggled to establish itself in England(15), but other collaborative ventures such as GP co-operatives were successful, particularly in improving GP job satisfaction(16, 17). A recent study of GP federations (voluntary collaborations between GP practices) found ‘successful’ federations were more likely to have coalesced around a particular service to be delivered or problem to be solved, and that collaborations took time to develop. Importantly, the study found that federations struggled with running costs, and required considerable managerial expertise to support them(18).

PCNs are an essential part of the delivery mechanisms for the Long Term Plan. They are also potentially crucial in the ongoing quest to stabilise general practice for the future. Evidence from previous collaborative ventures in primary care highlights an important issue: the way in which collaborations are established and the mechanisms used depend crucially upon the objectives being pursued. Thus, for example, a network with an educational objective requires a different structure to one by which incentive or other payments are to be distributed. It is therefore vital that we understand at this early stage what the underpinning and animating objectives for PCNs are

considered to be by those that are driving, or particularly connected to the development of, the policy as this will affect the approach required in their support and development.

In this paper we describe findings from the first phase of a longitudinal mixed-methods project tracking and exploring the development of PCNs and their associated outcomes. This phase of the project sought to understand in detail the national policy objectives associated with PCNs, and the mechanisms by which these objectives are expected to be achieved (‘programme theories’(19)). We show that the policy is underpinned by three broad groups of objectives. We then consider these alongside the content of the draft service specifications for PCNs issued late-December 2019(8), and note the revised specifications introduced as part of the updated GP contract published in February 2020(20). Our discussion explores the potential differences and synergies between the different objectives, and highlights issues which may need to be resolved.

Primary Care Networks: framework and funding

Forming or joining a PCN is voluntary, but practices are encouraged to engage by the provision of additional resources. The formal mechanisms by which practices work together is not prescribed, with guidance setting out a range of possible operating models with various implications associated with each(21). PCNs have been formed as a Directed Enhanced Service (DES), i.e. a nationally developed service and contractual addition to the core GMS/APMS/PMS primary care contract. The DES specification(22) requires that PCNs cover a 30-50,000 population (with some flexibilities) and be geographically contiguous.

Groups of practices were invited to apply to their local commissioner (Clinical Commissioning Group; CCG) to become a PCN from 1st July 2019. As part of the registration process PCN member practices signed a ‘network agreement’(23) outlining governance arrangements, including: the membership list, collective rights and obligations, financial entitlements. Additional detail regarding how practices could work with each other and with other organisations are set out in seven schedules attached to the agreement, although these were not required for initial registration. Each PCN identified a local clinician to be the Clinical Director (CD), which guidance(22) suggested would usually be a GP. CDs are required to work collaboratively with other CDs within the Integrated Care System (ICS)/Sustainability and transformation partnership (STP) area (inter-organisational partnerships between local councils and NHS organisations working to improve care across a system) and lead engagement with other local providers(22).

Once PCNs were approved by their CCG, they were eligible for the initial financial entitlements, which included: £1.50 per patient for participation, funded from the CCG core allocation; 0.25 full time equivalent per 50,000 population funding for the CD; funding for additional workforce roles. This latter was weighted according to the Carr Hill Formula which takes some account of deprivation and burden of morbidity. The initial focus was upon recruiting social prescribing link workers (funded at 100%) and clinical pharmacists (initially funded at 70%). PCNs also took over responsibility for providing extended access routine appointments during evenings and weekends; these were previously funded by a standalone DES payment to practices. There was no additional funding ear-marked for administrative or management costs.

The Network Contract DES will be renewed annually until 2024. The first year of the DES was framed as a development year for PCNs with the majority of service deliverables being monitored from 2020 onwards through additional service specifications(21). These were described in guidance as a key component of the DES and integral to supporting delivery of the NHS Long Term Plan(22). The first five specifications were intended to go live in April 2020: ‘Structured Medication Reviews and Medicines Optimisation’, ‘Enhanced Health in Care Homes’, ‘Anticipatory Care’, ‘Supporting Early

For peer review only

Cancer Diagnosis' and 'Personalised Care'. The final two specifications, 'Cardiovascular Disease Prevention and Diagnosis' and 'Tackling Neighbourhood Inequalities', were to be implemented from April 2021. There was little detail in the initial guidance of what the programmes of work would include, with details to be agreed as part of annual contract negotiations between the BMA and NHS England. Draft service specifications were published on the 23rd December 2019 alongside an engagement process which ended mid-January 2020. The initial draft was not well received by the GP profession at large(24), and in subsequent negotiations with the BMA significant concessions were made(20). These included: increasing funding for additional roles from 70% to 100%; increasing the range of roles eligible for reimbursement; reducing the number of service specifications to be delivered in the first year; and reducing the requirements for those specifications significantly in order to reduce the associated workload. It was suggested that, with the increase in funding for staff from 70% to 100%, the participation payment of £1.50 per patient would be released to fund administrative and managerial support. Further funding was also announced for a quality incentive scheme.

Methods

This paper presents findings from Work Package 1 of a longitudinal project exploring the development, operation, and outcomes associated with PCNs. Three other Work Packages are on-going or planned: a telephone survey of CCG PCN leads; in-depth qualitative PCN case studies; and a quantitative evaluation of PCN characteristics, activities, and outcomes.

Patient and public involvement

Due to the focus of this study, no patients were involved. The broader research project, within which this study sits, will explore the extent of patient and public involvement in PCN development and activity in a subsequent Work Package involving PCN case studies. A patient representative sits on the project advisory group and will continue to provide feedback to inform the development of the project.

Data collection, sampling and recruitment

Interviewees were purposively sampled for their knowledge about or role in the development of PCN policy. We sought participation from a range of relevant stakeholder organisations including NHS England and NHS Improvement, Department of Health and Social Care, and GP representative organisations. We undertook 16 semi-structured interviews with policy makers and stakeholders (07/2019-10/2019) by phone or face-to-face at interviewees' places of work. Interviews lasted between 30 and 60 minutes. The topic guide explored their interpretations of the objectives and associated mechanisms of the PCN policy. There was flexibility to explore interviewees' particular knowledge relating to their position or experience. Each interview was conducted by one of two experienced qualitative researchers (JH , KC), audio recorded and professionally transcribed. We also undertook a documentary analysis of a document published by the authority responsible for overseeing PCNs, NHS England, in December 2019 known as the 'draft service specifications'(8). This document set out in detail proposals for the work to be undertaken by PCNs. These draft proposals can be seen as an expression of the intended outcomes that senior policy makers wished PCNs to achieve, and our analysis involved testing the document against the themes derived from our interviews in order to better understand national policy objectives and their fit with those held by other stakeholders.

Data analysis

Transcripts were imported into NVivo 12 (QSR International) and analysed thematically. This process involved data familiarisation, open coding, theme identification, and review of themes. Coding and initial theme development was conducted by JH but the data and themes were reviewed by the

For peer review only

broader analysis team (JH, KC, LW-G) and refined iteratively over several rounds of analysis. The data constituting the themes was then additionally organised into a matrix by JH. Extracts were coloured by interviewee, similar extracts were grouped and connections to related extracts (mechanisms linked to particular outcomes, for example) visualised. This was used to aid decisions about what content relating to each theme should be included and emphasised in the findings below, with more frequently expressed perspectives featuring most prominently. These themes were them applied to the draft service specifications document(8).

Findings

We asked interviewees to explain what they thought the national policy objectives underlying PCNs were, and to describe potential or intended mechanisms. Interviewees were thus presenting their interpretations of policy objectives, not their personal beliefs about what might actually happen. We identified three main groups of objectives underlying the policy: use inter-GP practice collaboration to support a primary care sector which is struggling; align primary care more closely with other community services, improving integration and service delivery; and provide a collective 'voice' for primary care in the wider system. We explore and illustrate each of these in turn. Interview extracts are denoted by a unique participant ID code in square brackets (e.g. [N710cg]). We then consider the framing of policy objectives in the draft service specifications(8), and note a shift in this in the revised specifications in the updated GP contract(20).

Theme1: Supporting general practice

Within this theme, PCNs represent a vehicle for supporting general practice to reduce some of the pressures it currently faces in terms of unmanageable workloads, and related challenges recruiting and retaining sufficient GPs and nurses. The key mechanism for realising this objective is the new staff that PCNs will recruit through the Additional Roles Reimbursement Scheme (ARRS). Once in place, these staff are expected to reduce the workload burden on GPs, increasing work satisfaction and subsequently improving GP recruitment and retention rates. The consequence of this is to 'rescue' general practice from the pressures it faces and increase its resilience.

Resilience is also highlighted as an overarching benefit of collaborative working 'at scale.' This involves protection against negative consequences of shocks (both endogenous and exogenous) by virtue of operating as part of a larger inter-organisational entity. For example, one interviewee stated: "…networks provide an opportunity for greater resilience, so if a partner breaks their leg, the practice doesn't fall over"[N710cg]. In addition to protection for individual organisations, network membership was also expected to create other 'synergistic' benefits, such as new opportunities or increased efficiency, as a consequence of operating at a larger size. Examples offered to illustrate this included the ability to utilise clinical pharmacists across a collective footprint of networked practices when it would make little practical sense for any of those individual practices to employ a pharmacist for their patients alone.

It is the DES and associated financial incentives for GP practices that create the conditions for widespread PCN involvement. Respondents argued this collaboration would involve sharing of learning, data, and risk between practices, which would lead to improved inter-practice communication and the building of greater trust. An associated outcome was a reduction in intra-PCN variation as optimal approaches are identified and adopted by networked GP practices. This will result in improved patient experience as health care services become more accessible to patients and better tailored to local patient need. There were also expectations that reductions in inequalities locally could be mirrored nationally once the service specifications were introduced and best practices became established nationwide.

For peer review only

Theme 2: Place-based inter-organisational collaboration
The theme above is concerned with inter-GP practice collaboration, this theme is defined by an emphasis on inter-organisational collaborations between GP practices and other organisations and services in localities where PCNs are situated. One anticipated outcome is that more integrated and 'joined up' care will be delivered to patients in community settings. GP practices would forge closer connections to a range of local community resources and services, not just those directly health related, and more effectively and consistently direct patients towards them. Consequently, health care utilisation in general, and secondary care demand (including emergency admissions) in particular, would be reduced.

> "…aim is to bring together different providers in the primary care setting within networks, so within general practices, but also other providers and the voluntary sector and the community itself, to design and deliver services around specific needs of the community so to work in a networked way and try to achieve all the benefits that that would bring."[N800zf]

Interviewees recognised that it would be necessary to incentivise (non-GP) providers, such as community service providers, in order to facilitate their involvement in PCN activities towards fulfilling the aspirations of the policy, and this is planned through changes to, for example, the standard community services contract and pharmacy contract.

Some interviewees also suggested that PCNs were concerned with the development of an enhanced population health management approach whereby a range of health and other data relevant to local populations would be used to inform population segmentation, risk management assessments of particular groups, and the creation of multidisciplinary teams. This would deliver a new depth of understanding about local demography and health care related need.

Theme 3: Providing a 'voice' for primary care
This theme relates to PCNs' interaction with organisational entities in the broader system within which they are nested, and thus relates to both horizontal and vertical interactions rather than horizontal only. The Long Term Plan conceptualised the English NHS as a series of spatial tiers – neighbourhood, place, system – with PCNs operating at the neighbourhood level; CCGs, local councils, and hospitals at the place level; and ICSs/STPs at the system level. Interviewees framed PCNs as foundational building blocks for this spatial model, integral to supporting the levels above in their operation, or as an animating force that would bring life to arrangements. More specifically, PCN CD involvement at ICS/STP board level was highlighted as providing a means for PCNs to shape the development of the system of which they are a part and influence provider organisations at 'higher' levels. In doing so, CDs would provide a voice for primary care at system level and represent the interests of general practice and their PCN. This is made practically more feasible by the 'at scale' approach to general practice organisation associated with PCNs: "Having a stronger voice perhaps for general practice around those particular tables that hasn't always been possible or practical to do with practices working individually"[n210x8].

Summary
It is important to note that interviewees did not consider the objectives and mechanisms associated with each theme to necessarily be discrete or mutually exclusive. The majority primarily emphasised theme 1 'Supporting general practice', theme 2 'Place-based inter-organisational collaboration' to a lesser degree, and theme 3 'Providing a 'voice' for primary care' to a lesser degree still. One interviewee emphasised themes 1 and 3 largely equally but not theme 2. Four of the 16 interviewees gave similar weighting to the importance of all three themes. Interviewees from a background close to general practice were more likely to emphasise theme 1. Themes were also, in some cases,

For peer review only

considered to form a temporal sequence. Some interviewees talked about 'Supporting general practice' being the necessary first step before 'Place-based inter-organisational collaboration' could be more fully realised. However, others suggested it was only by GP practices working more closely with community service providers and third sector organisations that conditions in primary care would change to allow the workforce crisis to be addressed. Overall, whilst we have grouped the policy objectives into three overarching themes, it is clear that each was very broad, encompassing a significant number of potential objectives, mechanisms and expected outcomes. Our interviewees differed in how they envisaged the temporal sequencing of the desired objectives, and in the emphasis they placed on the different groups.

Draft service specifications

The draft service specifications were published on December 23rd 2019(8). Whilst the introduction to the document references reducing GP workload(8 p4), the focus within the specifications is upon the delivery of additional services by PCNs. Two of the five services (Structured Medication Reviews…, and Enhanced Care in Care Homes) were intended to be fully implemented from April 2020, with three more – Anticipatory Care; Personalised Care; Supporting Early Cancer Diagnosis - introduced from April 2020 in a phased manner over successive years until 2023/24 in order to avoid "…overburdening [PCNs] at an early stage with unrealistic expectations for new service delivery."(8 p4)

The specification document offers a clear programme theory for the policy:

> *"Though [sic] a combination of the additional workforce capacity within primary care, and the redesign of community services provision to link with and support PCNs, we expect the Network Contract DES both to reduce workload pressures on GPs and support improved primary care services to patients."(8 p4)*

It is also suggested that the additional workforce recruited will be sufficient to cover all work associated with delivering the five service specifications, whilst simultaneously providing spare capacity to take up some work currently undertaken by GPs. Thus, it is claimed, the workload burden on practices will reduce, although no evidence is provided to support this.

Structured medication reviews are to be delivered by individual practices, supported by clinical pharmacists. However, to perform these checks pharmacists will need prescribing qualifications, and not all pharmacists being recruited have this extra training. This work is therefore likely to devolve to GPs and any nurses with prescribing qualifications. Enhanced care in care homes will be delivered in collaboration with community service providers, as will anticipatory care, both of which require the establishment of network-level multidisciplinary teams. The personalised care service specification references better linkage with voluntary community groups, alongside the provision of personal health budgets. It is suggested this will enhance population health and reduce secondary care service use. Finally, the supporting earlier cancer diagnosis specification references greater collaboration between GPs and other service providers such as cancer alliances, secondary care and public health teams. The document explicitly references the intention that delivering the service specifications will lead to greater co-operation between GPs and community services, and suggests that this will be enhanced by forthcoming changes to the standard community services contract.

Taking the draft service specifications as a whole, the intention that PCNs will support the greater integration between primary care and community and other services comes through as the strongest underlying policy objective. References to practice workload are present, but only in so far as to make the argument that delivering these service specifications will have a beneficial effect on that workload, thereby supporting general practice. Little concrete evidence is provided to support

For peer review only

these arguments, beyond some general statements of mechanisms by which the services are expected to improve patients' health and therefore reduce demand.

The poor response from the profession to this document resulted in substantial changes during negotiations between the BMA and NHS England, including significantly increased funding, reduced requirements associated with the service specifications and increased flexibility in the ARRS(20). The document setting out the revised deal also offers a shift in tone towards our first theme, with a greater emphasis upon reducing workload for GPs(20).

Discussion

Our interviews illuminated a broad range of policy objectives to be achieved by PCNs, with three themes dominating the accounts: the need to support general practices; the desire to improve collaborative working between GP practices and other community-based services; and providing a collective voice for primary care within an evolving, more integrated, system. Importantly, we found our interviewees varied in terms of the emphasis they placed upon different groups of objectives, with those most closely linked to primary care tending to stress the need to support practices, whilst those more distant from practice more likely to focus upon the development of a more integrated system of community-based services. The objectives are clearly not mutually exclusive but may require temporal sequencing, with the stabilisation of a struggling primary care sector probably needing to occur before meaningful engagement with other community service providers can be achieved and before a 'collective voice' can be agreed. Moreover, different objectives imply different approaches to prioritising investment and may necessitate different incentives(25, 26), whilst approaches to providing support and training may differ according to which objectives are being pursued. For example, CDs will require different knowledge and skills to engage with ICSs than to work across practices to broker trust and engagement internally(27, 28).

Against this backdrop it is somewhat unsurprising that the draft service specifications released in December 2019 were not well received, as these almost exclusively focused upon the delivery of Long Term Plan objectives around joined up community-based services, with references to practice workload limited to arguments as to why delivery of these services will not increase workload. For those for whom the prime early objective of PCNs is to support general practice, such arguments are unlikely to be convincing. In subsequent negotiations the draft was changed significantly with greater emphasis upon practice workload and significantly increased funding(20). The manner in which this framing endures and evolves over-time is likely to have a bearing on the success of the implementation of the policy and warrants continued attention.

Strengths and limitations

This is an early study focusing upon policy objectives as perceived by those in senior positions across the system. As such, it inevitably offers a snapshot of a rapidly developing situation. Our wider study is ongoing and will explore the development of PCNs over time in more depth, as well as examining outcomes. We interviewed a relatively small number of people, however, participants were purposively selected to enable us to explore the interface between policy design and implementation as events unfolded, from the perspectives of some of those most closely involved.

Conclusion

Previous studies of GPs working together in a variety of ways have shown that collaborations can be associated with beneficial outcomes and that job satisfaction may increase(13, 16). However, the exact characteristics of particular collaborative ventures can have an impact on participation(26). The design of the PCN scheme and its embedded incentives therefore matters a great deal, and the policy context surrounding PCNs is moving rapidly. Our study offers an explanation for the problems

For peer review only

associated with the initial draft of the service specifications, going beyond a simple explanation based around workload to demonstrate a potential mismatch in objectives for the policy between different stakeholder groups. This offers policy makers some suggestions as to how confidence in the PCN policy might be restored in the longer term. All those we spoke to emphasised the general enthusiasm for collaboration between practices, and as we have highlighted such collaborations have a long history. Our findings suggest that early focus on measures to support practices may be rewarded by opportunities to deliver the broader integration agenda in the longer term. Slowing down the implementation of the policy may also be helpful.

More generally, our study highlights the importance of understanding the programme theories and objectives underlying policies. The PCN policy is wide ranging, and the successful establishment of PCNs may be able to offer multiple benefits to the health system. However, a failure to clarify the objectives behind the policy and a mismatch between the objectives of different groups may have played a part in the difficulties associated with the draft service specifications. Policy ambiguity has long been used by policy makers to enable the development of implementation coalitions(29, 30), but such ambiguity cannot be maintained in the face of concrete requirements for delivery. Our study confirms the need for those responsible for such requirements to first explore the range of objectives espoused by those who must act, and craft the delivery requirements in such a way that all groups can see some opportunity to meet their objectives(31).

Acknowledgements
The authors wish to thank the interviewees for their time and contribution to the study, and the broader PRUComm team.

Contributors
KC designed the study. JH and KC conducted the interviews relating to this paper. JH led the analysis of the data with support from KC, LW-G, SB. KC and JH drafted the manuscript to which all authors made substantial contributions. All authors approved the final version and agree to be accountable for all aspects of the analysis.

Funding
This research is part of independent research commissioned and funded by the NIHR Policy Research Programme (Ref: PR-PRU-1217-20801).

Disclaimer
The views expressed in this publication are those of the authors and do not necessarily reflect those of the Policy Research Programme, NIHR, NHS England, the Department of Health, arm's length bodies or other government departments.

Competing interests
The authors have no competing interests to declare.

Patient consent for publication
Not required.

Ethical approval
This study was granted ethical approval by The University of Manchester Proportionate Research Ethics Committee (study number: 2019-6922-11622). Participants were provided written information about the study, provided written consent or gave consent verbally at the beginning of telephone interviews.

For peer review only

Data sharing statement
No additional data are available.

References
1. Owen K, Hopkins T, Shortland T, Dale J. GP retention in the UK: a worsening crisis. Findings from a cross-sectional survey. BMJ Open. 2019;9(2):e026048.
2. Marchand C, Peckham S. Addressing the crisis of GP recruitment and retention: a systematic review. British Journal of General Practice. 2017;67(657):e227.
3. Iacobucci G. General practice funding rose by 3% last year. BMJ. 2017;358:j4367.
4. Charlesworth A, Bloor K. 70 years of NHS funding: how do we know how much is enough? BMJ. 2018;361:k2373.
5. NHS England. The General Practice Forward View. Leeds: NHS England; 2016.
6. Forbes LJ, Forbes H, Sutton M, Checkland K, Peckham S. How widespread is working at scale in English general practice? British Journal of General Practice. 2019.
7. NHS England. The NHS Long Term Plan. Leeds: NHS England; 2019.
8. NHS England and NHS Improvement. Network Contract Direct Enhanced Service:Draft Outline Service Specifications. Leeds: NHS England; 2019.
9. Swanson KA, Swanson JM, Gill AE, Walter C. Primary care in Cuba: A public health approach. Health Care for Women International. 1995;16(4):299-308.
10. Khoo HS, Lim YW, Vrijhoef HJ. Primary healthcare system and practice characteristics in Singapore. Asia Pacific family medicine. 2014;13(1):8.
11. Berridge V. Polyclinics: haven't we been there before? BMJ. 2008;336(7654):1161-2.
12. Humphrey C, Berrow D. Developing role of medical audit advisory groups. BMJ Quality & Safety. 1993;2(4):232-8.
13. Pickin D, O'cathain A, Fall M, Morgan A, Howe A, Nicholl J. The impact of a general practice co-operative on accident and emergency services, patient satisfaction and GP satisfaction. Family Practice. 2004;21(2):180-2.
14. Department of Health. Practice-based commissioning: engaging practices in commissioning. London: The Stationary Office; 2004.
15. Peckham S, Wilson P, Gosling J, Osipovic D, Wallace A. Community nursing in systems reform: the London polyclinic experience. British journal of community nursing. 2011;16(6):293-7.
16. Fletcher J, Pickard D, Rose J, Stewart-Brown S, Wilkinson E, Brogan C, et al. Do out-of-hours co-operatives improve general practitioners' health? Br J Gen Pract. 2000;50(459):815-6.
17. Coleman A, Checkland K, Harrison S, Dowswell G. Practice-based Commissioning: Theory, implementation and outcome. Final report. University of Manchester: National Primary Care Research and Development Centre; 2009.
18. McDonald R, Riste l, Bailey S, Bradley F, Hammond J, Spooner S, et al. Learning about and learning from GP Federations in the English NHS – an exploratory longitudinal study.: NIHR; in press.
19. Weiss CH. Evaluation. Upper Saddle River, NJ: Prentice-Hall; 1998.
20. British Medical Association, NHS England and NHS Improvement. Update to the GP contract agreement 2020/21 - 2023/24. Leeds: NHS England; 2020.
21. British Medical Association. The Primary Care Network Handbook. London: BMA; 2019.
22. NHS England. Network Contract Directed Enhanced Service. Leeds: NHS England; 2019.
23. NHS England, British Medical Association. Mandatory network agreement. Leeds: NHS England; 2019.
24. Iacobucci G. New primary care network contract won't work, GPs warn. BMJ. 2020;368:m39.
25. Mueller E. How to manage networks? The role of network attributes and incentives in network governance. International Journal of Entrepreneurship and Small Business. 2012;15(1):57-75.

26. de Stampa M, Vedel I, Bergman H, Novella J-L, Lapointe L. Fostering participation of general practitioners in integrated health services networks: incentives, barriers, and guidelines. BMC Health Services Research. 2009;9(1):48.
27. Storey J, Holti R, Hartley J, Marshall M, Matharu T. Clinical leadership through commissioning: Does it work in practice? Health Services Management Research. 2015;28(1-2):1-8.
28. Edmonstone JD. Whither the elephant?: the continuing development of clinical leadership in the UK National Health Services. The International Journal of Health Planning and Management. 2014;29(3):280-91.
29. Ettelt S, Mays N, Allen P. The Multiple Purposes of Policy Piloting and Their Consequences: Three Examples from National Health and Social Care Policy in England. Journal of Social Policy. 2014;FirstView:1-19.
30. Harrison S, Wood B. Designing Health Service Organization in the UK, 1968 to 1998: from Blueprint to Bright Idea and 'Manipulated Emergence'. Public Administration. 1999;77(4):751-68.
31. Bailey S, Checkland K, Hodgson D, Mcbride A, Elvey R, Parkin S, et al. The policy work of piloting: a case study in the English NHS. Social Science & Medicine. 2017;179:210-7.

For peer review only

BMJ Open

Exploring the multiple policy objectives for Primary Care Networks: a qualitative interview study with national policy stakeholders

Journal:	*BMJ Open*
Manuscript ID	bmjopen-2020-038398.R1
Article Type:	Original research
Date Submitted by the Author:	23-Apr-2020
Complete List of Authors:	Checkland, Kath; The University of Manchester, Division of Population Health, Health Services Research, and Primary Care; School of Health Sciences; Faculty of Biology, Medicine and Health<br>Hammond, Jonathan; University of Manchester, Division of Population Health, Health Services Research, and Primary Care; School of Health Sciences; Faculty of Biology, Medicine and Health<br>Warwick-Giles, Lynsey; The University of Manchester, Division of Population Health, Health Services Research, and Primary Care; School of Health Sciences; Faculty of Biology, Medicine and Health<br>Bailey, Simon; University of Kent, Centre for Health Services Studies
**Primary Subject Heading**:	Health policy
Secondary Subject Heading:	General practice / Family practice, Health policy, Qualitative research
Keywords:	Health policy < HEALTH SERVICES ADMINISTRATION & MANAGEMENT, QUALITATIVE RESEARCH, Organisation of health services < HEALTH SERVICES ADMINISTRATION & MANAGEMENT, PRIMARY CARE

SCHOLARONE™
Manuscripts

I, the Submitting Author has the right to grant and does grant on behalf of all authors of the Work (as defined in the below author licence), an exclusive licence and/or a non-exclusive licence for contributions from authors who are: i) UK Crown employees; ii) where BMJ has agreed a CC-BY licence shall apply, and/or iii) in accordance with the terms applicable for US Federal Government officers or employees acting as part of their official duties; on a worldwide, perpetual, irrevocable, royalty-free basis to BMJ Publishing Group Ltd ("BMJ") its licensees and where the relevant Journal is co-owned by BMJ to the co-owners of the Journal, to publish the Work in this journal and any other BMJ products and to exploit all rights, as set out in our licence.

The Submitting Author accepts and understands that any supply made under these terms is made by BMJ to the Submitting Author unless you are acting as an employee on behalf of your employer or a postgraduate student of an affiliated institution which is paying any applicable article publishing charge ("APC") for Open Access articles. Where the Submitting Author wishes to make the Work available on an Open Access basis (and intends to pay the relevant APC), the terms of reuse of such Open Access shall be governed by a Creative Commons licence – details of these licences and which Creative Commons licence will apply to this Work are set out in our licence referred to above.

Other than as permitted in any relevant BMJ Author's Self Archiving Policies, I confirm this Work has not been accepted for publication elsewhere, is not being considered for publication elsewhere and does not duplicate material already published. I confirm all authors consent to publication of this Work and authorise the granting of this licence.

For peer review only

Exploring the multiple policy objectives for Primary Care Networks: a qualitative interview study with national policy stakeholders

Authors

Kath Checkland, PhD, Professor of Health Policy and Primary Care, GP
Centre for Primary Care and Health Services Research
Division of Population Health, Health Service Research & Primary Care
School of Health Sciences
Faculty of Biology, Medicine and Health
The University of Manchester
Williamson Building, Oxford Road, Manchester, M13 9PL
Email: katherine.h.checkland@manchester.ac.uk
Twitter: @khcheck

Jonathan Hammond, PhD
Centre for Primary Care and Health Services Research
Division of Population Health, Health Service Research & Primary Care
School of Health Sciences
Faculty of Biology, Medicine and Health
The University of Manchester
Williamson Building, Oxford Road, Manchester, M13 9PL

Lynsey Warwick-Giles, PhD
Centre for Primary Care and Health Services Research
Division of Population Health, Health Service Research & Primary Care
School of Health Sciences
Faculty of Biology, Medicine and Health
The University of Manchester
Williamson Building, Oxford Road, Manchester, M13 9PL

Simon Bailey, PhD
Centre for Health Services Studies
University of Kent
Canterbury, Kent, CT2 7NF

For peer review only

Abstract

Objectives
English general practice is suffering a workforce crisis, with GPs retiring early and trainees reluctant to enter the profession. To address this, additional funding has been offered, but only through participation in collaborations known as Primary Care Networks (PCNs). This study explored national policy objectives underpinning PCNs, and mechanisms expected to help achieve these, from the perspective of those driving the policy.

Design
Qualitative semi-structured interviews and policy document analysis.

Setting and participants
National level policy maker and stakeholder interviewees (n=16). Policy document analysis of Network Contract Direct Enhanced Service draft service specifications.

Analysis
Interviews were transcribed, coded, and organised thematically according to policy objectives and mechanisms. Thematic data was organised into a matrix so prominent elements to be identified and emphasised accordingly. Themes were considered alongside objectives embedded in PCN draft service delivery requirements.

Results
Three themes of policy objectives and associated mechanisms were identified:(1) Supporting general practice;(2) Place-based inter-organisational collaboration;(3) Primary care 'voice'. Interviewees emphasised and sequenced themes differently suggesting meeting objectives for one was necessary to realise another. Interviewees most closely linked to primary care emphasised the importance of(1). The objectives embedded in draft service delivery requirements primarily emphasised(2).

Conclusions
These policy objectives are not mutually exclusive but may imply different approaches to prioritising investment or necessitate more explicit temporal sequencing, with the stabilisation of a struggling primary care sector probably needing to occur before meaningful engagement with other community service providers can be achieved or a 'collective voice' is agreed. Multiple objectives create space for stakeholders to feel dissatisfied when implementation details do not match expectations, as the negative reaction to draft service delivery requirements illustrates. Our study offers policy makers suggestions about how confidence in the policy might be restored by crafting delivery requirements so all groups see opportunities to meet favoured objectives.

Keywords
health policy; qualitative research; organisation of health services; primary care

For peer review only

Strengths and limitations of this study

- Primary Care Networks represent a significant policy development in England, and we offer the first systematic analysis of national policy objectives as articulated by a range of different stakeholders.
- We interviewed 16 national level policy makers or stakeholders working in a range of organisations, including NHS England and NHS Improvement, and in government in the Department of Health and Social Care.
- National health care policy objectives are rarely subject to critical academic attention in the early stages of policy implementation, but doing so provides scope for better understanding challenges a policy may face and developing strategies to address these.
- This is a fast moving policy area and our results inevitably reflect a particular snapshot of time.

Main text: 4218 words

For peer review only

Introduction

Primary care in the UK is in crisis. Young doctors are not entering, or remaining in, the speciality in sufficient numbers to cope with demand, many older GPs are increasingly dissatisfied with general practice due to various intrinsic and extrinsic factors and choosing to retire early (1, 2). The problem is complex, arising from the intersection between demographic change, rising population expectations of what services should deliver, and funding growth which has not matched the rise in costs(3, 4). In keeping with this complexity, potential solutions are multiple, with, for example, the GP Forward View(5) offering a number of potential policy fixes. Most recently, consensus has gathered around the idea that GP practices – traditionally independent contractors to the NHS – should work together more effectively to support one another and provide a broader range of services(6). To enable this, a centrepiece of the new NHS Long Term Plan(7) is the creation and funding of Primary Care Networks (PCNs). The underlying idea is relatively simple: incentivise GP practices to combine together into groups, so they can find economies of scale, employ a wider range of staff, link more effectively with community-based providers and, through this, improve services to patients.

Collaborations between GP practices are not new, in the UK or elsewhere. Internationally a 'polyclinic' model is said to offer the advantages of person-centred primary care alongside an extended range of services from a multidisciplinary team(8-10). In the UK, practices have voluntarily formed themselves into collaborative groups to undertake collective audits(11), deliver out-of-hours services(12) and support commissioning(13). Outcomes from these previous collaborations have been mixed. The 'polyclinic' model struggled to establish itself in England(14), but other collaborative ventures such as GP co-operatives were successful, particularly in improving GP job satisfaction(15, 16). A recent study of GP federations (voluntary collaborations between GP practices) found 'successful' federations were more likely to have coalesced around a particular service to be delivered or problem to be solved, and that collaborations took time to develop. Importantly, the study found that federations struggled with running costs, and required considerable managerial expertise to support them(17).

PCNs are an essential part of the delivery mechanisms for the Long Term Plan. They are also potentially crucial in the ongoing quest to stabilise general practice for the future. Evidence from previous collaborative ventures in primary care highlights an important issue: the way in which collaborations are established and the mechanisms used to establish and support them depend crucially upon the objectives being pursued. Thus, for example, a network with an educational objective requires a different structure to one by which incentive or other payments are to be

distributed. It is therefore vital that we understand at this early stage what the underpinning and animating objectives for PCNs are considered to be by those that are driving, or particularly connected to the development of, the policy, as this will affect the rules, funding mechanisms and support put in place. Whilst the perspectives of policy makers are likely to differ from those more closely involved with frontline general practice, their perspectives are important because they determine the formal policy mechanisms which govern PCN operation.

In this paper we describe findings from the first phase of a longitudinal mixed-methods project tracking and exploring the development of PCNs and their associated outcomes. This phase of the project sought to understand in detail the national policy objectives associated with PCNs, and the mechanisms by which these objectives are expected to be achieved ('programme theories'(18)). We show that the policy is underpinned by three broad groups of objectives. We then consider these alongside the content of the draft service specifications for PCNs issued late-December 2019(19), and note the revised specifications introduced as part of the updated GP contract published in February 2020(20). Our discussion explores the potential differences and synergies between the different objectives, and highlights issues which may need to be resolved.

Primary Care Networks: framework and funding

Forming or joining a PCN is voluntary, but practices are encouraged to engage by the provision of additional resources. The formal mechanisms by which practices work together is not prescribed, with guidance setting out a range of possible operating models with various implications associated with each(21). PCNs have been formed as a Directed Enhanced Service (DES), i.e. a nationally developed service and contractual addition to the core GMS/APMS/PMS primary care contract. The DES specification(22) requires that PCNs cover a 30-50,000 population (with some flexibilities) and be geographically contiguous.

Groups of practices were invited to apply to their local commissioner (Clinical Commissioning Group; CCG) to become a PCN from 1st July 2019. As part of the registration process PCN member practices signed a 'network agreement'(23) outlining governance arrangements, including: the membership list, collective rights and obligations, financial entitlements. Additional detail regarding how practices could work with each other and with other organisations are set out in seven schedules attached to the agreement, although these were not required for initial registration. Each PCN identified a local clinician to be the Clinical Director (CD), which guidance(22) suggested would usually be a GP. CDs are required to work collaboratively with other CDs within the Integrated Care System (ICS)/Sustainability and transformation partnership (STP) area (inter-organisational partnerships between local councils and NHS organisations working to improve care across a system) and lead engagement with other local providers(22).

Once PCNs were approved by their CCG, they were eligible for the initial financial entitlements, which included: £1.50 per patient for participation, funded from the CCG core allocation; 0.25 full time equivalent per 50,000 population funding for the CD; funding for additional workforce roles. This latter was weighted according to the Carr Hill Formula which takes some account of deprivation and burden of morbidity. The initial focus was upon recruiting social prescribing link workers (funded at 100%) and clinical pharmacists (initially funded at 70%). PCNs also took over responsibility for providing extended access routine appointments during evenings and weekends; these were previously funded by a standalone DES payment to practices. There was no additional funding ear-marked for administrative or management costs.

The Network Contract DES will be renewed annually until 2024. The first year of the DES was framed as a development year for PCNs with the majority of service deliverables being monitored from 2020

onwards through additional service specifications(21). These were described in guidance as a key component of the DES and integral to supporting delivery of the NHS Long Term Plan(22). The first five specifications were intended to go live in April 2020: 'Structured Medication Reviews and Medicines Optimisation', 'Enhanced Health in Care Homes', 'Anticipatory Care', 'Supporting Early Cancer Diagnosis' and 'Personalised Care'. The final two specifications, 'Cardiovascular Disease Prevention and Diagnosis' and 'Tackling Neighbourhood Inequalities', were to be implemented from April 2021. There was little detail in the initial guidance of what the programmes of work would include, with details to be agreed as part of annual contract negotiations between the BMA and NHS England. Draft service specifications were published on the 23rd December 2019 alongside an engagement process which ended mid-January 2020. The initial draft was not well received by the GP profession at large(24), and in subsequent negotiations with the BMA significant concessions were made(20). These included: increasing funding for additional roles from 70% to 100%; increasing the range of roles eligible for reimbursement; reducing the number of service specifications to be delivered in the first year; and reducing the requirements for those specifications significantly in order to reduce the associated workload. It was suggested that, with the increase in funding for staff from 70% to 100%, the participation payment of £1.50 per patient would be released to fund administrative and managerial support. Further funding was also announced for a quality incentive scheme. A number of concerns about the policy have been expressed (25, 26) . In particular, commentators have highlighted the very broad range of activity that PCNs are expected to engage in, and the lack of evidence underpinning some of this activity. Refining and improving the policy over time will require constructive engagement with the official objectives underlying the particular framework which has been established. This study offers the first empirical evidence of what those objectives are.

Methods

This paper presents findings from Work Package 1 of a longitudinal project exploring the development, operation, and outcomes associated with PCNs. Three other Work Packages are on-going or planned: a telephone survey of CCG PCN leads; in-depth qualitative PCN case studies; and a quantitative evaluation of PCN characteristics, activities, and outcomes.

Patient and public involvement

Due to the focus of this study, no patients were involved. The broader research project, within which this study sits, will explore the extent of patient and public involvement in PCN development and activity in a subsequent Work Package involving PCN case studies. A patient representative sits on the project advisory group and will continue to provide feedback to inform the development of the project.

Data collection, sampling and recruitment

Interviewees were purposively sampled for their knowledge about or role in the development of PCN policy. We sought participation from a range of relevant stakeholder organisations including NHS England and NHS Improvement, Department of Health and Social Care, and GP representative organisations. We undertook 16 semi-structured interviews with policy makers and stakeholders (07/2019-10/2019) by phone or face-to-face at interviewees' places of work. Interviews lasted between 30 and 60 minutes. The topic guide explored their interpretations of the objectives and associated mechanisms of the PCN policy. There was flexibility to explore interviewees' particular knowledge relating to their position or experience. Each interview was conducted by one of two experienced qualitative researchers (JH , KC), audio recorded and professionally transcribed.

We also undertook a documentary analysis of a document published by the authority responsible for overseeing PCNs, NHS England, in December 2019 known as the 'draft service specifications'(8). This

For peer review only

document set out in detail proposals for the work to be undertaken by PCNs. These draft proposals can be seen as an expression of the intended outcomes that senior policy makers wished PCNs to achieve, and our analysis involved testing the document against the themes derived from our interviews in order to better understand national policy objectives and their fit with those held by other stakeholders.

Data analysis

Transcripts were imported into NVivo 12 (QSR International) and analysed thematically. This process involved data familiarisation, open coding, theme identification, and review of themes. Coding and initial theme development was conducted by JH but the data and themes were reviewed by the broader analysis team (JH, KC, LW-G) and refined iteratively over several rounds of analysis. The data constituting the themes was then additionally organised into a matrix by JH. Extracts were coloured by interviewee, similar extracts were grouped and connections to related extracts (mechanisms linked to particular outcomes, for example) visualised. This was used to aid decisions about what content relating to each theme should be included and emphasised in the findings below, with more frequently expressed perspectives featuring most prominently. Data collection and analysis took place in parallel and the thematic matrix demonstrated clearly that data saturation was reached during our final interviews. Themes were then applied in the analysis of the draft service specifications document(8).

Findings

We asked interviewees to explain what they thought the national policy objectives underlying PCNs were, and to describe potential or intended mechanisms. Interviewees were thus presenting their interpretations of policy objectives, not their personal beliefs about what might actually happen. We recognise that national policy makers and stakeholders may have particular perspectives about the state and needs of general practice that differ from those of others working in different parts of the system in different capacities. Our intention here is not to adjudicate between these perspectives, but to present the perspectives of those responsible for developing and implementing the rules and funding mechanisms which govern PCN operation.

We identified three main groups of objectives espoused by those with senior-level responsibility for implementing or shaping the policy: use inter-GP practice collaboration to support a primary care sector which is struggling; align primary care more closely with other community services, improving integration and service delivery; and provide a collective 'voice' for primary care in the wider system. We explore and illustrate each of these in turn. Interview extracts are denoted by a unique participant ID code in square brackets (e.g. [N710cg]). We then consider the framing of policy objectives in the draft service specifications(19), and note a shift in this in the revised specifications in the updated GP contract(20).

Theme1: Supporting general practice

Within this theme, PCNs represent a vehicle for supporting general practice to reduce some of the pressures it currently faces in terms of unmanageable workloads, and related challenges recruiting and retaining sufficient GPs and nurses. The key mechanism for realising this objective is the new staff that PCNs will recruit through the Additional Roles Reimbursement Scheme (ARRS). Once in place, these staff are expected to reduce the workload burden on GPs, increasing work satisfaction and subsequently improving GP recruitment and retention rates. The consequence of this is to 'rescue' general practice from the pressures it faces and increase its resilience.

Resilience is also highlighted as an overarching benefit of collaborative working 'at scale.' This involves protection against negative consequences of shocks (both endogenous and exogenous) by

For peer review only

virtue of operating as part of a larger inter-organisational entity. For example, one interviewee stated: "…networks provide an opportunity for greater resilience, so if a partner breaks their leg, the practice doesn't fall over"[N710cg]. In addition to protection for individual organisations, network membership was also expected to create other 'synergistic' benefits, such as new opportunities or increased efficiency, as a consequence of operating at a larger size. Examples offered to illustrate this included the ability to utilise clinical pharmacists across a collective footprint of networked practices when it would make little practical sense for any of those individual practices to employ a pharmacist for their patients alone, or the sharing of back office functions across a larger footprint.

It is the DES and associated financial incentives for GP practices that create the conditions for widespread PCN involvement. Respondents argued this collaboration would involve sharing of learning, data, and risk between practices, which would lead to improved inter-practice communication and the building of greater trust. An associated outcome was a reduction in intra-PCN variation as optimal approaches are identified and adopted by networked GP practices. This will result in improved patient experience as health care services become more accessible to patients and better tailored to local patient need. There were also expectations that reductions in inequalities locally could be mirrored nationally once the service specifications were introduced and best practices became established nationwide.

Theme 2: Place-based inter-organisational collaboration

The theme above is concerned with inter-GP practice collaboration, this theme is defined by an emphasis on inter-organisational collaborations between GP practices and other organisations and services in localities where PCNs are situated. One anticipated outcome is that more integrated and 'joined up' care will be delivered to patients in community settings. GP practices would forge closer connections to a range of local community resources and services, not just those directly health related, and more effectively and consistently direct patients towards them. Consequently, health care utilisation in general, and secondary care demand (including emergency admissions) in particular, would be reduced.

> "…aim is to bring together different providers in the primary care setting within networks, so within general practices, but also other providers and the voluntary sector and the community itself, to design and deliver services around specific needs of the community so to work in a networked way and try to achieve all the benefits that that would bring."[N800zf]

Interviewees recognised that it would be necessary to incentivise (non-GP) providers, such as community service providers, in order to facilitate their involvement in PCN activities towards fulfilling the aspirations of the policy, and this is planned through changes to, for example, the standard community services contract and pharmacy contract.

Some interviewees also suggested that PCNs were concerned with the development of an enhanced population health management approach whereby a range of health and other data relevant to local populations would be used to inform population segmentation, risk management assessments of particular groups, and the creation of multidisciplinary teams. This would deliver a new depth of understanding about local demography and health care related need.

Theme 3: Providing a 'voice' for primary care

This theme relates to PCNs' interaction with organisational entities in the broader system within which they are nested, and thus relates to both horizontal and vertical interactions rather than horizontal only. The Long Term Plan conceptualised the English NHS as a series of spatial tiers – neighbourhood, place, system – with PCNs operating at the neighbourhood level; CCGs, local

For peer review only

councils, and hospitals at the place level; and ICSs/STPs at the system level. Interviewees framed PCNs as foundational building blocks for this spatial model, integral to supporting the levels above in their operation, or as an animating force that would bring life to arrangements. More specifically, PCN CD involvement at ICS/STP board level was highlighted as providing a means for PCNs to shape the development of the system of which they are a part and influence provider organisations at 'higher' levels. In doing so, CDs would provide a voice for primary care at system level and represent the interests of general practice and their PCN. This is made practically more feasible by the 'at scale' approach to general practice organisation associated with PCNs: "Having a stronger voice perhaps for general practice around those particular tables that hasn't always been possible or practical to do with practices working individually"[n210x8].

Summary

It is important to note that interviewees did not consider the objectives and mechanisms associated with each theme to necessarily be discrete or mutually exclusive. The majority primarily emphasised theme 1 'Supporting general practice', theme 2 'Place-based inter-organisational collaboration' to a lesser degree, and theme 3 'Providing a 'voice' for primary care' to a lesser degree still. One interviewee emphasised themes 1 and 3 largely equally but not theme 2. Four of the 16 interviewees gave similar weighting to the importance of all three themes. Interviewees from a background close to general practice were more likely to emphasise theme 1. Themes were also, in some cases, considered to form a temporal sequence. Some interviewees talked about 'Supporting general practice' being the necessary first step before 'Place-based inter-organisational collaboration' could be more fully realised. However, others suggested it was only by GP practices working more closely with community service providers and third sector organisations that conditions in primary care would change to allow the workforce crisis to be addressed. Overall, whilst we have grouped the policy objectives into three overarching themes, it is clear that each was very broad, encompassing a significant number of potential objectives, mechanisms and expected outcomes. Our interviewees differed in how they envisaged the temporal sequencing of the desired objectives, and in the emphasis they placed on the different groups.

Draft service specifications

The draft service specifications were published on December 23rd 2019(19). Whilst the introduction to the document references reducing GP workload(19 p4), the focus within the specifications is upon the delivery of additional services by PCNs. Two of the five services (Structured Medication Reviews…, and Enhanced Care in Care Homes) were intended to be fully implemented from April 2020, with three more – Anticipatory Care; Personalised Care; Supporting Early Cancer Diagnosis - introduced from April 2020 in a phased manner over successive years until 2023/24 in order to avoid "…overburdening [PCNs] at an early stage with unrealistic expectations for new service delivery."(19 p4)

The specification document offers a clear programme theory for the policy:

> *"Though [sic] a combination of the additional workforce capacity within primary care, and the redesign of community services provision to link with and support PCNs, we expect the Network Contract DES both to reduce workload pressures on GPs and support improved primary care services to patients."(19 p4)*

It is also suggested that the additional workforce recruited will be sufficient to cover all work associated with delivering the five service specifications, whilst simultaneously providing spare capacity to take up some work currently undertaken by GPs. Thus, it is claimed, the workload burden on practices will reduce, although no evidence is provided to support this.

For peer review only

Structured medication reviews are to be delivered by individual practices, supported by clinical pharmacists. However, to perform these checks pharmacists will need prescribing qualifications, and not all pharmacists being recruited have this extra training. This work is therefore likely to devolve to GPs and any nurses with prescribing qualifications. Enhanced care in care homes will be delivered in collaboration with community service providers, as will anticipatory care, both of which require the establishment of network-level multidisciplinary teams. The personalised care service specification references better linkage with voluntary community groups, alongside the provision of personal health budgets. It is suggested this will enhance population health and reduce secondary care service use. Finally, the supporting earlier cancer diagnosis specification references greater collaboration between GPs and other service providers such as cancer alliances, secondary care and public health teams. The document explicitly references the intention that delivering the service specifications will lead to greater co-operation between GPs and community services, and suggests that this will be enhanced by forthcoming changes to the standard community services contract.

Taking the draft service specifications as a whole, the intention that PCNs will support the greater integration between primary care and community and other services comes through as the strongest underlying policy objective. References to practice workload are present, but only in so far as to make the argument that delivering these service specifications will have a beneficial effect on that workload, thereby supporting general practice. Little concrete evidence is provided to support these arguments, beyond some general statements of mechanisms by which the services are expected to improve patients' health and therefore reduce demand.

The poor response from the profession to this document resulted in substantial changes during negotiations between the BMA and NHS England, including significantly increased funding, reduced requirements associated with the service specifications and increased flexibility in the ARRS(20). The document setting out the revised deal also offers a shift in tone towards our first theme, with a greater emphasis upon reducing workload for GPs(20).

Discussion

Previous studies of GPs working together in a variety of ways have shown that collaborations can be associated with beneficial outcomes and that job satisfaction may increase(13, 16). However, the exact characteristics of particular collaborative ventures can have an impact on participation(26). The design of the PCN scheme and its embedded incentives therefore matters a great deal, and the policy context surrounding PCNs is moving rapidly. Our interviews illuminated a broad range of policy objectives to be achieved by PCNs, with three themes dominating the accounts: the need to support general practices; the desire to improve collaborative working between GP practices and other community-based services; and providing a collective voice for primary care within an evolving, more integrated, system. Importantly, we found our interviewees varied in terms of the emphasis they placed upon different groups of objectives, with those most closely linked to primary care tending to stress the need to support practices, whilst those more distant from practice more likely to focus upon the development of a more integrated system of community-based services. The objectives are clearly not mutually exclusive but may require temporal sequencing, with the stabilisation of a struggling primary care sector probably needing to occur before meaningful engagement with other community service providers can be achieved and before a 'collective voice' can be agreed (25, 26) . Moreover, different objectives imply different approaches to prioritising investment and may necessitate different incentives(27, 28), whilst approaches to providing support and training may differ according to which objectives are being pursued. For example, CDs will require different knowledge and skills to engage with ICSs than to work across practices to broker trust and engagement internally(29, 30).

For peer review only

Against this backdrop it is somewhat unsurprising that the draft service specifications released in December 2019 were not well received, as these almost exclusively focused upon the delivery of Long Term Plan objectives around joined up community-based services, with references to practice workload limited to arguments as to why delivery of these services will not increase workload. For those for whom the prime early objective of PCNs is to support general practice, such arguments are unlikely to be convincing. In subsequent negotiations the draft was changed significantly with greater emphasis upon practice workload and significantly increased funding(20). The manner in which this framing endures and evolves over-time is likely to have a bearing on the success of the implementation of the policy and warrants continued attention.

Strengths and limitations

This is an early study focusing upon policy objectives as perceived by those in senior positions across the system. As such, it inevitably offers a snapshot of a rapidly developing situation. Our wider study is ongoing and will explore the development of PCNs over time in more depth, as well as examining outcomes. We interviewed a relatively small number of people, however, participants were purposively selected to enable us to explore the interface between policy design and implementation as events unfolded, from the perspectives of some of those most closely involved.

Conclusion

By exploring how policy makers and key stakeholders conceptualise the objectives of the PCN policy, and the variation between them, this study offers an explanation for the problems associated with the initial draft of the service specifications, going beyond a simple explanation based around workload to demonstrate a potential mismatch in objectives for the policy between different stakeholder groups. This offers policy makers some suggestions as to how confidence in the PCN policy might be restored in the longer term. All those we spoke to emphasised the general enthusiasm for collaboration between practices, and as we have highlighted such collaborations have a long history. Our findings suggest that early focus on measures to support practices may be rewarded by opportunities to deliver the broader integration agenda in the longer term. Slowing down the implementation of the policy may also be helpful.

More generally, our study highlights the importance of understanding the programme theories and objectives underlying policies. The PCN policy is wide ranging, and the successful establishment of PCNs may be able to offer multiple benefits to the health system. However, a failure to clarify the objectives behind the policy and a mismatch between the objectives of different groups may have played a part in the difficulties associated with the draft service specifications. Policy ambiguity has long been used by policy makers to enable the development of implementation coalitions(31, 32), but such ambiguity cannot be maintained in the face of concrete requirements for delivery. Furthermore, choosing a contractual mechanism for implementation (rather than, for example, a piloting approach using incentives) necessarily constrains local implementation discretion, and places limits on what can be done. A national contract with fixed funding models and standard delivery requirements implies certainty about what activity is required, something which is rarely present in health and care contracting (33, 34). Such a contract limits opportunities for PCNs to respond to local contextual conditions or to choose their own priorities, and this may be problematic in the longer term. At the very least, our study confirms the need for those responsible for setting contract requirements to understand the range of objectives espoused by those who must act to implement the policy, and suggests that delivery requirements should be crafted in such a way that all groups can see some opportunity to meet their objectives with flexibility to allow adaptation to local needs(35).

For peer review only

Acknowledgements

The authors wish to thank the interviewees for their time and contribution to the study, and the broader PRUComm team.

Contributors

KC designed the study. JH and KC conducted the interviews relating to this paper. JH led the analysis of the data with support from KC, LW-G, SB. KC and JH drafted the manuscript to which all authors made substantial contributions. All authors approved the final version and agree to be accountable for all aspects of the analysis.

Funding

This research is part of independent research commissioned and funded by the NIHR Policy Research Programme (Ref: PR-PRU-1217-20801).

For peer review only

Disclaimer

The views expressed in this publication are those of the authors and do not necessarily reflect those of the Policy Research Programme, NIHR, NHS England, the Department of Health, arm's length bodies or other government departments.

Competing interests

The authors have no competing interests to declare.

Patient consent for publication

Not required.

Ethical approval

This study was granted ethical approval by The University of Manchester Proportionate Research Ethics Committee (study number: 2019-6922-11622). Participants were provided written information about the study, provided written consent or gave consent verbally at the beginning of telephone interviews.

Data sharing statement

No additional data are available.

References

1. Owen K, Hopkins T, Shortland T, Dale J. GP retention in the UK: a worsening crisis. Findings from a cross-sectional survey. BMJ Open. 2019;9(2):e026048.
2. Marchand C, Peckham S. Addressing the crisis of GP recruitment and retention: a systematic review. British Journal of General Practice. 2017;67(657):e227.
3. Iacobucci G. General practice funding rose by 3% last year. BMJ. 2017;358:j4367.
4. Charlesworth A, Bloor K. 70 years of NHS funding: how do we know how much is enough? BMJ. 2018;361:k2373.
5. NHS England. The General Practice Forward View. Leeds: NHS England; 2016.
6. Forbes LJL, Forbes H, Sutton M, Checkland K, Peckham S. How widespread is working at scale in English general practice? An observational study. British Journal of General Practice. 2019;69(687):e682.
7. NHS England. The NHS Long Term Plan. Leeds: NHS England; 2019.

8. Swanson KA, Swanson JM, Gill AE, Walter C. Primary care in Cuba: A public health approach. Health Care for Women International. 1995;16(4):299-308.
9. Khoo HS, Lim YW, Vrijhoef HJ. Primary healthcare system and practice characteristics in Singapore. Asia Pacific family medicine. 2014;13(1):8.
10. Berridge V. Polyclinics: haven't we been there before? BMJ. 2008;336(7654):1161-2.
11. Humphrey C, Berrow D. Developing role of medical audit advisory groups. BMJ Quality & Safety. 1993;2(4):232-8.
12. Pickin D, O'cathain A, Fall M, Morgan A, Howe A, Nicholl J. The impact of a general practice co-operative on accident and emergency services, patient satisfaction and GP satisfaction. Family Practice. 2004;21(2):180-2.
13. Department of Health. Practice-based commissioning: engaging practices in commissioning. London: The Stationary Office; 2004.
14. Peckham S, Wilson P, Gosling J, Osipovic D, Wallace A. Community nursing in systems reform: the London polyclinic experience. British journal of community nursing. 2011;16(6):293-7.
15. Fletcher J, Pickard D, Rose J, Stewart-Brown S, Wilkinson E, Brogan C, et al. Do out-of-hours co-operatives improve general practitioners' health? Br J Gen Pract. 2000;50(459):815-6.
16. Coleman A, Checkland K, Harrison S, Dowswell G. Practice-based Commissioning: Theory, implementation and outcome. Final report. University of Manchester: National Primary Care Research and Development Centre; 2009.
17. McDonald R, Riste l, Bailey S, Bradley F, Hammond J, Spooner S, et al. Learning about and learning from GP Federations in the English NHS – an exploratory longitudinal study.: NIHR; in press.
18. Weiss CH. Evaluation. Upper Saddle River, NJ: Prentice-Hall; 1998.
19. NHS England and NHS Improvement. Network Contract Direct Enhanced Service:Draft Outline Service Specifications. Leeds: NHS England; 2019.
20. British Medical Association, NHS England and NHS Improvement. Update to the GP contract agreement 2020/21 - 2023/24. Leeds: NHS England; 2020.
21. British Medical Association. The Primary Care Network Handbook. London: BMA; 2019.
22. NHS England. Network Contract Directed Enhanced Service. Leeds: NHS England; 2019.
23. NHS England, British Medical Association. Mandatory network agreement. Leeds: NHS England; 2019.
24. Iacobucci G. New primary care network contract won't work, GPs warn. BMJ. 2020;368:m39.
25. Wilson T, Lewis R. Primary care networks: well intentioned but overambitious. BMJ. 2019;366:l5311.
26. Murray R. GPs condemn new specifications for primary care networks. BMJ. 2020;368:m258.
27. Mueller E. How to manage networks? The role of network attributes and incentives in network governance. International Journal of Entrepreneurship and Small Business. 2012;15(1):57-75.
28. de Stampa M, Vedel I, Bergman H, Novella J-L, Lapointe L. Fostering participation of general practitioners in integrated health services networks: incentives, barriers, and guidelines. BMC Health Services Research. 2009;9(1):48.
29. Storey J, Holti R, Hartley J, Marshall M, Matharu T. Clinical leadership through commissioning: Does it work in practice? Health Services Management Research. 2015;28(1-2):1-8.
30. Edmonstone JD. Whither the elephant?: the continuing development of clinical leadership in the UK National Health Services. The International Journal of Health Planning and Management. 2014;29(3):280-91.
31. Ettelt S, Mays N, Allen P. The Multiple Purposes of Policy Piloting and Their Consequences: Three Examples from National Health and Social Care Policy in England. Journal of Social Policy. 2014;FirstView:1-19.
32. Harrison S, Wood B. Designing Health Service Organization in the UK, 1968 to 1998: from Blueprint to Bright Idea and 'Manipulated Emergence'. Public Administration. 1999;77(4):751-68.

For peer review only

33. Allen P. A socio-legal and economic analysis of contracting in the NHS internal market using a case study of contracting for district nursing. Social science and medicine. 2002;54(2):255-66.
34. Schwartz R. The Contracting Quandary: Managing Local Authority–VNPO Relations. Local Government Studies. 2005;31(1):69-83.
35. Bailey S, Checkland K, Hodgson D, Mcbride A, Elvey R, Parkin S, et al. The policy work of piloting: a case study in the English NHS. Social Science & Medicine. 2017;179:210-7.

For peer review only
